# Characterization of Fish Gelatin Obtained from Atlantic Cod Skin Using Enzymatic Treatment

**DOI:** 10.3390/polym14040751

**Published:** 2022-02-15

**Authors:** Svetlana R. Derkach, Daria S. Kolotova, Yuliya A. Kuchina, Nadezhda V. Shumskaya

**Affiliations:** 1 Laboratory of Chemistry and Technology of Marine Bioresources, Institute of Natural Science and Technology, Murmansk State Technical University, Sportivnaya Str., 13, 183010 Murmansk, Russia; derkachsr@mstu.edu.ru (S.R.D.); kuchinayua@mstu.edu.ru (Y.A.K.); 2 Laboratory of Aquaculture and Aquatic Diseases, Polar Branch of Russian Research Institute of Fishiries and Oceanography, Academician Knipovich Str., 6, 183038 Murmansk, Russia; shumskaya@pinro.ru

**Keywords:** fish gelatin, enzymatic treatment, gelatin extraction, functional properties, hydrogels, rheological properties

## Abstract

In recent years, there has been increased interest in the production of gelatin from alternative sources, such as raw fish materials. Traditionally, gelatin is obtained using an acidic or alkaline treatment. However, these methods have some disadvantages, such as the long times for processing raw materials and the use of large amounts of water and chemicals. Furthermore, milder processing regimes are required for producing fish gelatin. Enzymes could be the solution for improving the technology of fish gelatin production, due to their specificity and ability to increase the rate of collagen digestion. In this work, samples of gelatin from cod skin were obtained using enzymes of bacterial (protosubtilin) and animal (pancreatin) origins. The use of enzymes reduced the duration of extraction by 40%, and the yield of the final product was increased from 51% to 58–60%. The dependence of the contents of the main components of the secondary structure of gelatin and its rheological and thermal properties on molecular weight was also established. In this study, the gelatins obtained without enzymes and with protosubtilin were shown to have the most desirable characteristics, namely of the highest molecular weights and the highest proportion of ordered structures.

## 1. Introduction

Gelatin is a protein compound obtained as a result of the digestion of native collagen contained in the bones, cartilage, and skin of mammals and fish [1]. The type of raw material used and its chemical composition are important factors affecting the properties of gelatin [2,3,4]. The most common sources of gelatin for industrial production are pork and bovine skins and the bones of pigs and cattle.

However, in recent years, there has been a significant increase in the production of gelatin from alternative sources, such as raw fish materials [5,6,7,8,9]. In addition to the well-known sociocultural and veterinary-sanitary aspects [10], the existing need for the rational utilization of waste from the fishing industry is one of the reasons for the increased interest in fish gelatin. The catch of cold water fish from the seas of the Northern Basin is increasing every year, which makes them an attractive raw material for obtaining gelatin. Of particular interest is Atlantic cod, which is a valuable commercial species due to its wide range of habitats and its nutritional properties. Cod has a high content of essential amino acids and has a balanced protein–lipid composition. In recent years, the catch of cod in the Northern Basin has increased by 4%, amounting to about 400 thousand tons per year [11]. At the same time, a significant portion of the catch is sent for processing to obtain skinned fillets, which leads to the accumulation of a large number of collagen-containing byproducts. The yield of collagen-containing waste (skin, scales, bones, fins) during deep cutting of raw fish materials represents 35 to 60% of the total fish mass [12]. A significant content of collagen is contained in the skin of the fish, comprising up to about 90%.

The collagen molecule (tropocollagen) is a left-handed helix composed of three α-chains linked by covalent bonds. The primary structure of the protein is built of repetitive Gly–X–Y chains, where X is usually proline, and Y is often hydroxyproline [13]. The short N- and C-terminal regions do not form triple helices since they mainly consist of lysine and hydroxylysine residues and their derivatives, which are linked by both intra- and intermolecular covalent cross-links [14].

The transition of collagen to gelatin is a process in which a highly organized, water-insoluble collagen fiber transforms from an endless asymmetric network of interconnected tropocollagen units into simpler structural units with a lower degree of internal order. To break noncovalent bonds between α-chains and disorganize the protein structure, a pre-chemical acidic or alkaline treatment is usually used [15]. Then, the gelatin extraction stage is carried out in an aqueous medium under the influence of temperature. At this stage, the splitting of hydrogen and covalent bonds occurs, by which the collagen’s triple helix is irreversibly denatured, resulting in the formation of three polypeptide α-chains that are gelatin molecules [16,17].

The degree of collagen conversion to gelatin depends on the stage of pretreatment and on the conditions of the extraction process (pH, temperature, and extraction time) [18]. Depending on the stage of pretreatment, two types of gelatins are distinguished: type A gelatin (the isoelectric point at pH 7–9) and type B gelatin (the isoelectric point at pH 4–5), obtained under acidic or alkaline pretreatment, respectively.

The main disadvantages of both acidic and alkaline treatments of collagen are the long processing times for collagen-containing raw materials, the use of large amounts of water, and the use of chemical reagents. The water and reagent use leads to the formation of large amounts of waste and, consequently, to serious environmental problems [19,20]. In the production of fish gelatin, mild and sparing processing modes are required, along with a short extraction time, weakly acidic environment, and a temperature regime that does not cause cleavage of gelatin α-chains.

The use of enzymes in the production of gelatin from collagen-containing raw fish materials is one of the promising ways to improve the technological process [21]. Due to their specificity, enzymes have targeted effects on certain bonds in the collagen molecule and help to increase the rate of digestion [22].

As a rule, enzymes of natural origins are used to obtain gelatin. The most widespread are commercial proteases from plant sources, such as papain, and proteases of animal origins, such as pepsin and trypsin [12]. Pancreatin, a commercial food-grade enzyme with high proteolytic activity, is also used to obtain gelatin [23].

Enzymes of microbial origin are also used to obtain gelatin from raw fish materials. In comparison with enzymes of animal or plant origins, microbial enzymes have higher levels of catalytic activity and wider operating ranges of pH and temperature for which the enzyme remains stable [24]. For example, it has been shown that Alcalase 2, 4 L can significantly accelerate the gelatin extraction process in comparison with the use of neutral or acidic enzymes [25]. A more advanced approach was patented, which uses proteolytic enzymes produced by nonpathogenic bacteria [26]. It was shown that the enzyme preparation protosubtilin has higher collagenase activity in the temperature range 35–45 °C compared to papain, which exhibits maximum activity at temperatures close to the temperature of protein denaturation [27].

When obtaining gelatin from raw fish materials, issues related to satisfactorily increasing product yield and decreasing the time required for technological operations remain unresolved. The need to preserve the physicochemical and functional properties of fish gelatin while improving technologies is an important factor. To address these points, in this work, an enzymatic method for obtaining gelatin from the skin of Atlantic cod was developed and trialed.

Commercially available enzyme pancreatin and enzyme preparation protosubtilin, which have high proteolytic activity, were chosen for enzyme-based gelatin preparations. We chose those because, firstly, they have optimal activity at relatively low temperatures (~40 °C) that do not cause protein denaturation and deterioration in the functional properties of gelatin, so the energy costs for gelatin production are reduced. Secondly, protosubtilin and pancreatin exhibit maximal activity at neutral pH, which can significantly reduce the number of reagents required to regulate the pH of the medium. Finally, they are safe and can be used to produce food-grade fish gelatin.

Thus, the purpose of this work was to improve the technology used for obtaining fish gelatin by using enzymes with proteolytic action to reduce the time taken for gelatin extraction and increase the yield without losing functional properties.

## 2. Materials and Methods

### 2.1. Materials

The skin of medium-sized (70–90 cm long) Atlantic cod (*G. morhua*) was used for gelatin extraction. Atlantic cod was caught by Trawl Fleet Co., Ltd. (Murmansk, Russia) in the Barents Sea and delivered chilled to the port of Murmansk. The skin was manually cleaned of scales and muscle fragments using a knife, thoroughly washed in tap water, frozen, and stored at −20 °C before the use.

Gelatin from cold-water fish skin G 7041 (Sigma, Oakville, ON, Canada) was used as a standard sample.

Protosubtilin G3x (Sibbiopharm, Berdsk, Russia) and pancreatin (ICN Biochemicals, Pittsburgh, PA, USA) were used as enzymes to obtain gelatin.

Protosubtilin is an enzyme preparation of bacterial origin, which is a mixture of enzymes (neutral and alkaline proteinases, alpha-amylase, beta-glucanase, xylanase, and cellulase). Maximum activity is manifested at pH 6.5–7.5 and 40–55 °C.

Pancreatin is an enzyme obtained from the pancreases of mammals. It contains protease, amylase, and lipase. Its optimum action is achieved at pH 7.4–8.5 in the temperature range of 40–50 °C.

### 2.2. Gelatin Extraction

Frozen cod skin was defrosted, washed with tap water, cut into pieces of 0.5 cm × 0.5 cm, mixed with distilled water at a mass ratio of 1:6, and incubated at a temperature of 20 ± 1 °C with constant stirring for 1 h The mixture was then filtered through 3 layers of gauze. Filter cake (collagen-containing raw materials) was mixed with distilled water at a mass ratio of 1:4. Extraction of gelatin was carried out at pH = 7.0–7.5, temperature 40 ± 1 °C for 3 h. The concentration of the enzyme was 0.025 g/kg of raw material. The reaction mixture was then heated to 85–90 °C for 5 min to inactivate the enzyme. The mixture was then filtered through a filter cloth (belting) and centrifuged in a rotary centrifuge (Beckman Coulter, Inc., Bray, CA, USA) at a speed of 5000 rpm for 40 min at a temperature of 20.0 ± 0.5 °C. The gelatin solution was dried in a FreeZone freeze dryer (Labconco, South Kansas City, MO, USA) at a temperature of −50 °C and a residual pressure of 2.4–2.6 Pa. The resulting gelatin was ground into powder and stored at a temperature not exceeding 5 °C until further use. The samples obtained will be further designated in accordance with Table 1.

The yield of gelatin (B, %) was calculated using Formula (1):

(1) B=(m/M) × 100%,

where *m*—weight of dry gelatin, g; *M*—weight of dried raw material with the moisture content not exceeding 10%, g.

### 2.3. Chemical Composition of Gelatin

The chemical compositions (moisture content, content of fat, protein substances (total nitrogen), and mineral substances) of gelatin samples were determined according to standard methods [28]. The moisture content in a sample was determined after it was dried to a constant weight at t = 105 ± 5 °C. The amount of fat was determined using the Soxhlet method of solvent extraction. Protein content was determined using the Kjeldahl method (P = N_total_·5.55). The amine nitrogen was determined using the formol titration method. The content of mineral substances (ash) was determined by burning the sample in an electric muffle at a temperature of 550 ± 10 °C.

### 2.4. Amino Acid Composition

The amino acid composition of gelatin was determined using high-performance liquid chromatography. The analysis was performed using an LC-20 AD Prominence system (Shimadzu, Kyoto, Japan) with a reaction module for post-column derivatization with ninhydrin ARM-1000 (Sevko & Co, Moscow, Russia) and an ion-exchange resin column 4.6 mm × 150 mm (Sevko & Co, Russia). Beforehand, acid hydrolysis of the samples was carried out in 6N HCl for 24 h at a temperature of 110 ± 2 °C. The resulting solution of free amino acids was dried and then dissolved in sodium citrate buffer solution (Sevko & Co, Moscow, Russia). Prior to being introduced into the chromatographic system, the samples were filtered using a membrane filter (Agilent, Santa Clara, CA, USA) with a pore diameter of 0.45 µm. Standard buffer solutions for sample dilution and elution (Sevko & Co, Moscow, Russia) were used. The concentration was calculated using a standard amino acid sample (Sykam, Eresing, Germany).

### 2.5. Molecular Weight

The molecular weight distribution of each gelatin sample was determined using high-performance liquid chromatography (HPLC). Peaks were recorded with an LCMS-QP8000 gas chromatography mass spectrometer (Shimadzu, Kyoto, Japan) with a spectrophotometric detector (SPD-10 AVVP) at λ = 280 nm. Chromatographic separation was carried out on a Tosoh TSKgel Alpha-4000 column at 25 °C in an isocratic mode with the flow rate of 0.8 mL/min; the eluent was an aqueous solution of NaCl with a concentration of 0.15 mol/L and the sample volume was 10 μL. Proteins of known molecular weights from Sigma-Aldrich (St. Louis, MI, USA) [29] were used to calibrate the column. The correlation coefficient was 0.97. The average molecular weight (M_w_, kDa) was calculated using the equation of the linear relationship between the detector response and the decimal logarithm of the molecular weight:

(2) $${\mathrm{lgM}}_{w}=-0.6012\;\cdot \;V+10.126,$$

where V—volume of eluent passed through the column (cm^3^).

### 2.6. Secondary Structure of Gelatin

IR spectroscopy was used to evaluate the secondary structures of the gelatin macromolecules. Absorption spectra were recorded on an IRTracer-100 Fourier transform infrared spectrophotometer (Shimadzu, Kyoto, Japan) in the frequency range of 4000–800 cm^−1^, at a resolution of 4 cm^−1^ (number of scans 250).

Each sample used for research was a mixture of gelatin and KBr in a weight ratio of 1:120. The mixture was dissolved in distilled water and then dried in a freeze dryer at −50 °C and a residual pressure of 2.4–2.6 Pa for 8–10 h. The dried mixture was additionally kept in a drying oven at a temperature of 60 ± 5 °C for 6 h. The mixture (m = 150 mg) was then compressed under a pressure of 650 kgf/cm^2^ in the form of a tablet. IR spectra were recorded immediately after pressing. The water vapor spectrum was subtracted from the obtained spectra to minimize the effect of traces of moisture (water vapor).

The IR spectrum in the absorption region of the amide I band (1600–1700 cm^−1^) was processed using OriginPro 9.0 (OriginLab Corporation, Northampton, MA, USA). Analysis of the secondary structures of gelatin macromolecules was carried out using the method of the second derivative. The amide I band was decomposed into several components with the use of Gaussian distribution to obtain quantitative information [30,31]. The quantitative contribution of each component of the secondary structure was determined as the ratio of the integrated intensity of the corresponding band to the total integrated intensity of the amide I band before decomposition.

### 2.7. Emulsification Capability

For the oil–water emulsions, 4% aqueous solutions of fish gelatin were used as the respective aqueous phases, and refined sunflower oil (MEZ Yug Rusi Ltd., Rostov-on-Don, Russia) was used as the oil phase. The oil–water ratio varied from 10:90 to 90:10 (vol%/vol%). Emulsions were prepared by dispersing oil in water at room temperature using an Ultra Turrax T25 Digital (IKA-Werke, Staufen im Breisgau, Germany) equipped with a S25N18G shaft. Dispersion speed was 8000 rpm, and dispersion time was 10 min. The freshly prepared emulsions were placed in graduated cylinders, and their separation was observed for 96 h at room temperature. The emulsifying ability of fish gelatin samples and the stability of the resulting emulsions were characterized using the emulsion index (EI, %). EI is the ratio of the emulsion formed after 96 h (V_em_) to the total volume of liquid in the cylinder (V_total_) [32]:

(3) $$\mathrm{EI}=\frac{V_{\mathrm{em}}}{V_{\mathrm{total}}}\times 100\%$$

### 2.8. Rheological Tests

The rheological properties of gelatin gels were measured using an MCR 302 modular compact rheometer (Anton Paar, Graz, Austria) equipped with a cone-and-plate working unit. The diameter of the cone was 50 mm, and the angle between the surface of the cone and the plate was 1°; the distance between the cone apex and the plate was 0.100 mm. Gelatin gels with a concentration of 10% for rheological measurements were prepared according to the following protocol. The gelatin was preliminarily swollen in distilled water for 1 h at room temperature. Then the gelatin was dissolved at room temperature and with constant stirring. Gels were obtained by cooling aqueous solutions of gelatin at a temperature of 6 °C for 24 h.

The following experimental protocols for measurements were applied [33]:

- Periodic oscillations with a constant frequency of ω = 6.28 rad/s, and amplitude sweep changes in the range from 0.01% to 1000%.
- Periodic oscillations with a constant amplitude of γ = 1% (corresponding to the domain of linear viscoelasticity), and a frequency sweep in the range from 0.01 to 500 s^−1^.
- Temperature scanning with a step size of 1 °C/min at a constant frequency of 6.28 rad/s (1 Hz) and constant amplitude of deformation of γ = 1%.
- Time dependence of the elastic modulus at 6 °C, at a constant frequency of ω = 6.28 rad/s and constant amplitude of deformation of γ = 1% (for following the kinetics of gelation); the initial temperature was 25 °C.
- Isothermal creep and elastic recovery at 6 °C and constant stress in the range of 5–300 Pa for loading for 15 min and recovery for 15 min.

## 3. Results

### 3.1. Chemical Composition

The chemical compositions of the fish gelatin samples are shown in Table 2.

As can be seen in Table 2, the obtained samples were characterized by high protein content, reaching values of 92.0–94.4%, and insignificant content of mineral substances, not exceeding 1.1%. All samples did not contain fats, and the moisture content did not exceed 6.8 wt.%, which is an acceptable value for commercial gelatin [34]. All samples were white or light-beige powders. Analysis of the data in Table 2 shows that the type of enzyme did not significantly affect the chemical composition of the obtained gelatin sample. However, the use of enzymes to produce gelatin made it possible to reduce the duration of the extraction stage by 40% (from 5 to 3 h), whereas the yield of the final product increased from 51% to 58–60%.

### 3.2. Amino Acid Composition 

Table 3 shows the amino acid compositions of the obtained fish gelatin samples. The analysis shows that all samples, regardless of the extraction conditions, were characterized by high contents of glycine, alanine, proline, arginine, serine, and aspartic and glutamic acids. However, the contents of essential amino acids tyrosine and methionine in the obtained samples decreased by 25% and 60%, respectively, compared to the initial raw material. In addition, in gelatin samples obtained under the action of enzymes, the amounts of glycine and alanine decreased (for G2, roughly by 2.5% and 9.0%, respectively; for G3, roughly by 4.2% and 4.0%, respectively), in comparison with the sample obtained without enzymes. Gelatin samples obtained using enzymes (G2 and G3) were characterized by reduced contents of proline (~40%) and methionine (~55%) and increased contents of arginine (~40–45%), aspartic acid (~20–25%), and lysine (two times) compared to the commercial sample G 7041.

### 3.3. Molecular Weight

**Figure 1:**
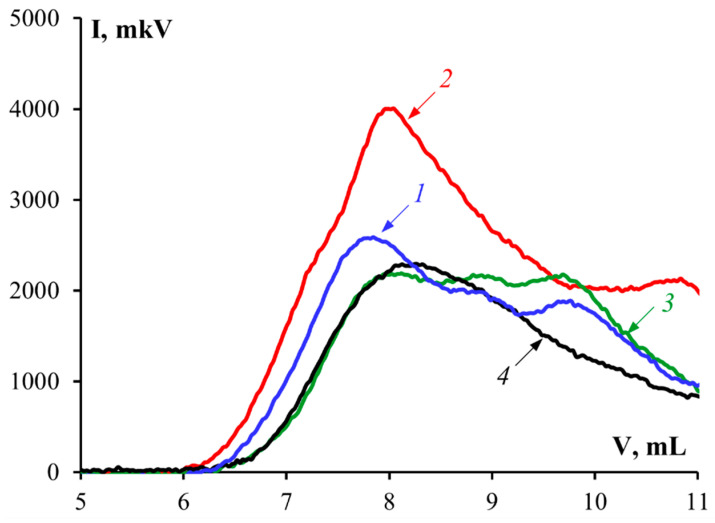
Chromatograms of gelatin samples: 1—G1, 2—G2, 3—G3, 4—G 7041.

Molecular weight distribution is an important indicator of gelatin quality, which determines the physicochemical and functional properties of gelatin. Figure 1 shows chromatograms characterizing the molecular weight distributions of gelatin samples obtained from the cod skin and G 7041, the standard sample.

All gelatin samples contained several fractions corresponding to different molecular weight ranges (Figure 1). The chromatograms contain two peaks: the first corresponds to protein fractions with molecular weights of about 160–200 kDa, and the second to 40–70 kDa. In this case, the first fraction was the main contributor in determining the average molecular weight (M_w_) (Table 4). For gelatin obtained using enzyme preparations (samples G2 and G3), there was a slight shift of the peaks toward low M_w_ values in comparison to gelatin obtained without enzymes. Gelatin obtained using protosubtilin (G2) had a broader molecular weight distribution. According to HPLC data (Figure 1, Table 4), gelatin samples obtained using protosubtilin (G2) and pancreatin (G3) had lower average molecular weights (M_w_ ≈ 138.9 and 97.1 kDa, respectively) compared to sample G1 obtained without enzymes (M_w_ ≈ 158.8 kDa). It is possible that when enzymes are used for gelatin extraction, denaturation of the collagen triple helix occurs together with the formation of α and β-chains, and the hydrolysis of α-chains into lower molecular weight fragments also takes place. Under the action of protosubtilin, this process proceeds more slowly, which is indicative of the low collagenase activity of this enzyme preparation. The standard sample of gelatin G 7041 had an average molecular weight of 131.5 kDa.

### 3.4. Secondary Structures of the Gelatin Macromolecules

The IR spectrum of gelatin, as a protein, is characterized by the presence of several main absorption bands corresponding to vibrational transitions in the peptide chain [35,36]. Table 5 shows the main absorption bands of the functional groups of gelatin.

**Figure 2:**
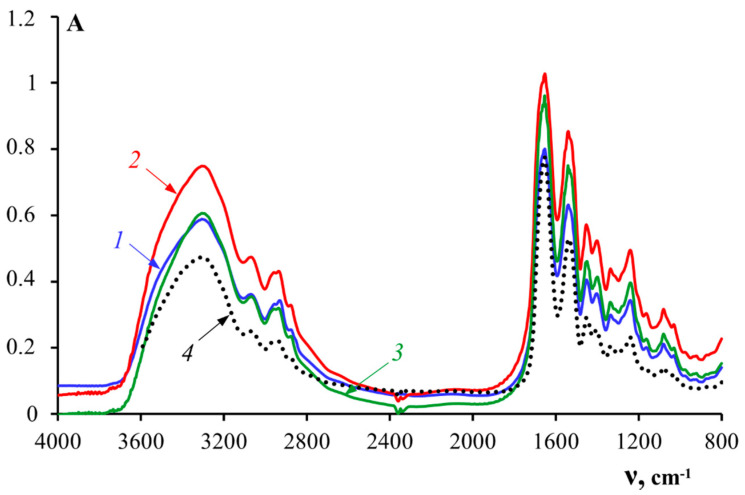
FTIR spectra of gelatin samples: 1—G1, 2—G2, 3—G3, 4—G 7041.

Figure 2 shows the FTIR spectra of fish gelatin samples. A standard fish gelatin sample, Sigma, was used as a control. Analysis of FTIR spectra showed that a change in the extraction conditions did not lead to a shift in the peaks. These IR spectra are typical for fish gelatin and are comparable with the results obtained for gelatin from other fish species [37,38].

The amide I band is most sensitive to changes in the secondary structure of gelatin. It corresponds to the stretching vibrations of the C=O bonds of the polypeptide chain. The amide I band has a complex contour, which is qualitatively explained by the superposition of bands corresponding to different conformational states of the polypeptide chain. The IR spectra of gelatin were analyzed using the method of the second derivative in the absorption region of the amide I band to obtain information about the secondary structure of gelatin. The absorption bands in the spectral range of amide I were compared with various conformational states of gelatin, based on the literature data (Table 6) [30,31,39].

**Figure 3:**
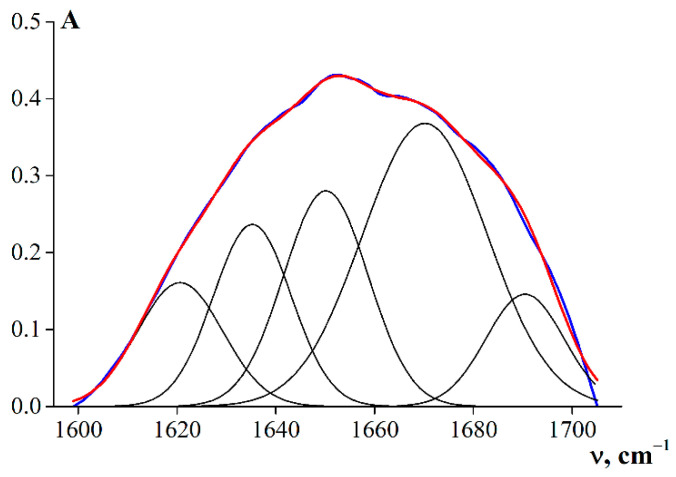
Decomposition of the band components of the amide I band using a Gaussian distribution (R^2^ = 0.99).

The integral intensities of the corresponding peaks were determined to obtain quantitative information on the fractions of the macromolecular chain of gelatin in various conformations. For this purpose, the amide I band was decomposed using a Gaussian distribution. Figure 3 shows an example of the graphical decomposition of an amide I band into the corresponding theoretically obtained Gaussians for sample G2.

Table 6 shows the results of the theoretical decompositions of the amide I bands into Gaussians corresponding to different conformational states of the macro-chains of fish gelatin samples.

Analysis of the data in Table 6 shows that gelatins obtained using enzymes (samples G2 and G3) were characterized by more random structures (random coil) and fewer ordered structures (triple helices), as compared to the sample obtained without enzymes (G1) [40]. It is important to note there was greater denaturation of helices under the action of pancreatin, as indicated by the changes in the triple helix content by up to 40.6% (G2) and 22.4% (G3), in comparison with gelatin obtained without the enzyme (G1), for which the triple helix content was 42.9%.

**Figure 4:**
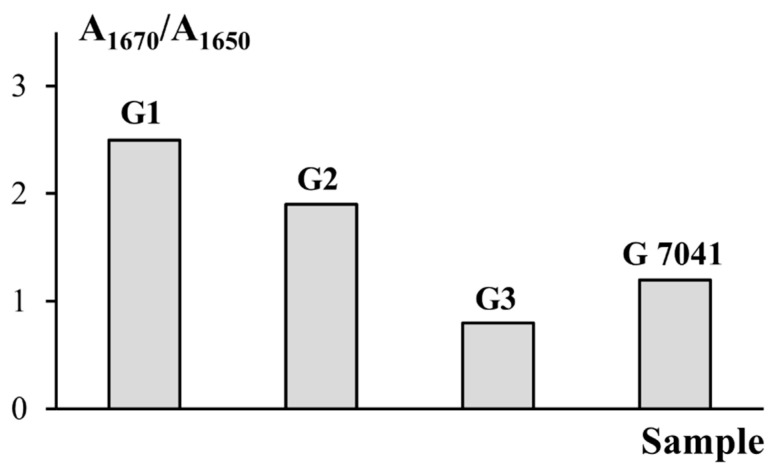
The ratios of the integral intensity of the peaks corresponding to triple helixes (A1670) and random coils (A1650) in the second derivative spectra of fish gelatin.

These findings are supported by data for the molecular weight distributions of the gelatin samples (Figure 1, Table 4). The ratio of the integrated intensity of the peaks corresponding to α-helixes (triple helix) to that of the peaks corresponding to coils (random coil) was used to assess the triple helix content of the gelatin samples (Figure 4). These data are correlated with the results of the molecular weight distribution of gelatin (Table 4). Thus, samples with high contents of triple helices had higher molecular weights. The degree of denaturation under the action of pancreatin was higher than under the action of protosubtilin.

### 3.5. Emulsification Capability

**Figure 5:**
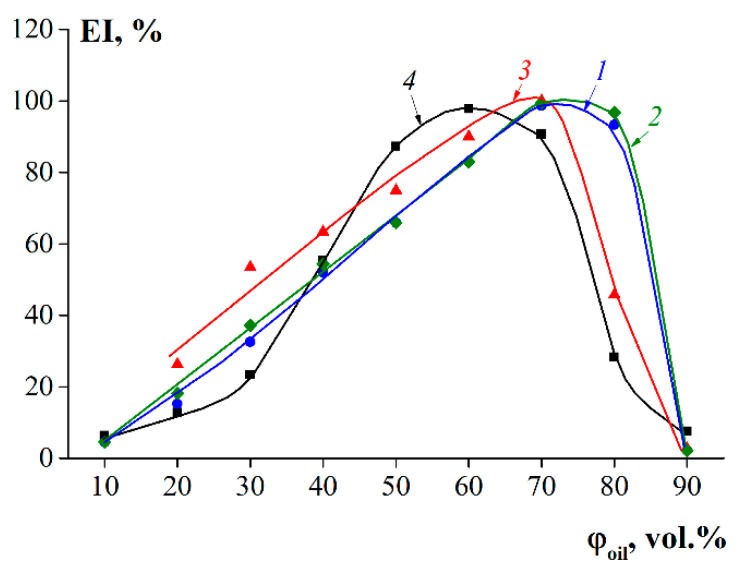
The dependences of emulsion index (EI) on oil volume fractions for emulsions stabilized with fish gelatin, where 1—G1, 2—G2, 3—G3, and 4—G 7041. Gelatin concentration was 4 wt.%.

The emulsion index (EI) reflects the ability of gelatin molecules to adsorb at the interface and resist the instability of emulsions, e.g., manifesting a creaming, coalescence, or flocculation. Figure 5 shows the dependences of each EI for the oil–water emulsions stabilized with fish gelatin on the volume fraction of the respective oil phase. The most stable emulsions (EI ~100%) for samples G1, G2, and G3 could be obtained at an oil–water ratio of 70:30 (vol.%/vol.%). For fish gelatin G 7041, this ratio was 60:40 (vol.%/vol.%) when the EI was 91%. Figure 5 shows that gelatin samples G1 and G2 had the highest stabilizing ability, since they formed stable oil–water emulsions, even at an oil–water ratio of 80:20 (vol.%/vol.%).

### 3.6. Thermal Properties of Gelatin

Gelatin macromolecules are capable of a thermoreversible conformational transition, random coil → triple helix, in aqueous systems. When the concentration of gelatin exceeds a certain critical concentration, this process is accompanied by a thermoreversible transition from sol to gel. The quality of gelatin for a specific application is mainly determined by its rheological properties, such as gel strength and thermal stability, which are characterized by the melting temperature (T_m_) and the gelation temperature (T_g_) [41].

**Figure 6:**
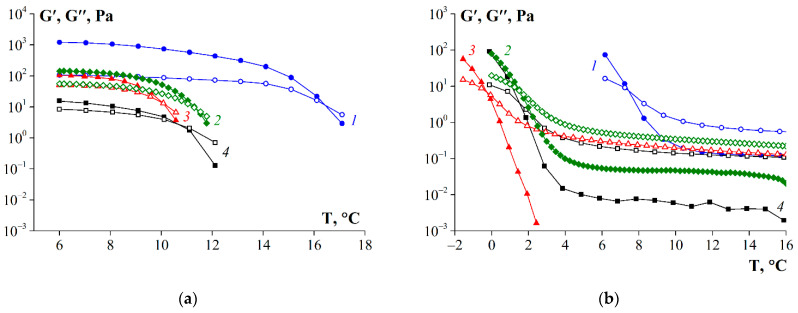
Storage (G′, closed symbols) and loss (G″, open symbols) modulus’s dependency on temperature obtained during the heating (**a**) and cooling (**b**) at ω = 6.28 and γ = 1 %∙rad/s. Scanning rate was 1 °C/min. 1—G1, 2—G2, 3—G3, 4—G 7041. Gelatin concentration was 10 wt.%.

Figure 6 shows a comparison of the dynamic viscoelastic profiles of fish gelatin samples obtained in heating (Figure 6a) and cooling (Figure 6b) modes in the temperature range from −2 to 18 °C with a temperature change rate of 1 °C/min. During cooling, the values of storage modulus G′ sharply increase upon reaching a certain temperature as a result of the formation of bonding zones and the strengthening of the structural network of the gel. In this case, the maximum values of dynamic moduli at higher temperatures were observed for sample G1, which indicates a tendency to form triple helixes [17]. Sample G3 had the lowest values of storage modulus and melting and gelation points, which are related to low thermal stability. The behavior of sample G2 was similar to the standard sample of fish gelatin G 7041, but G2 was characterized by higher values of storage modulus and melting point. As a rule, higher values of storage modulus and melting/gelation temperature of gelatin are characteristic of samples with high molecular weights and high contents of proline and hydroxyproline, which play a unique role in the stabilization of triple helices [17].

Based on the data shown in Figure 6, the values of T_m_ and T_g_ were determined as the intersections of the dependences of the dynamic modules on the temperature obtained in the heating and cooling modes, respectively. As can be seen in Table 7, sample G1 was characterized by the highest values of melting and gelation temperatures. The smallest values of T_m_ and T_g_ were observed for sample G3 that, most likely, was subject to the highest degree of collagen hydrolysis due to the specificity of the enzyme for the raw material used. The properties of sample G2 were similar to those of the standard sample, G 7041.

### 3.7. Rheological Properties of Gelatin Gels

**Figure 7:**
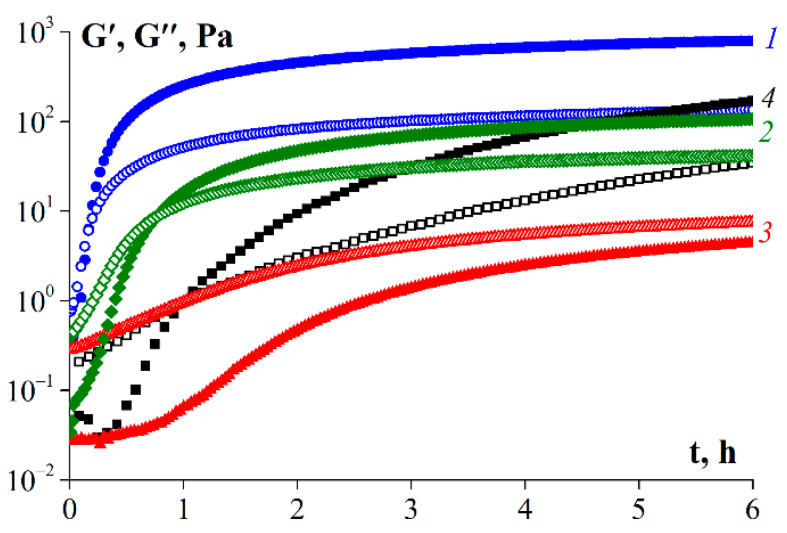
Gelation kinetics of gelatin samples: 1—G1, 2—G2, 3—G3, 4—G 7041. T = 6 °C, ω = 6.28 rad/s, γ = 1%. Gelatin concentration was 10 wt.%. The initial temperature of the samples was 25 °C.

Figure 7 shows the gelation kinetic curves for the fish gelatin samples. The sample obtained without enzyme (G1) had the highest gelation rate, as evidenced by the rapid increase in the storage modulus over time. In this case, the characteristic gelation time required to achieve constant values of storage modulus was approximately 4 h. The gelatin obtained using protosubtilin (G2) had a high gelation rate than G 7041, and their gelation times were 5 and 6 h, respectively. For the sample obtained using pancreatin (G3), gel formation was not observed even within 6 h, as evidenced by the predominance of the loss modulus over the storage modulus in the entire studied time range.

**Figure 8:**
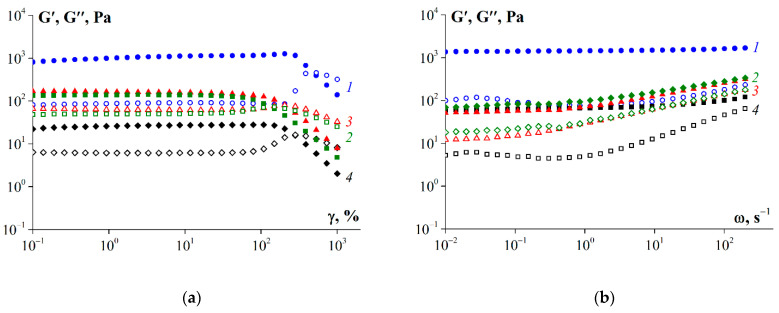
(**a**) Storage (G′, closed symbols) and loss (G″, open symbols) moduli dependency on amplitude at 6 °C and ω = 6.28 rad/s; (**b**) G′ (closed symbols) and G″ (open symbols) moduli dependency on frequency at 6 °C and γ = 1 %. 1—G1, 2—G2, 3—G3, 4—G 7041. Gelatin concentration was 10 wt.%.

Figure 8 shows the amplitude dependences of the dynamic moduli of fish gelatin gels, obtained at 6 °C. The storage modulus exceeded the loss modulus in all gelatin sample. This behavior is characteristic of the solid state of the material. Sample G1 was characterized by the highest values of storage modulus, which indicates the higher “rigidity” of this gel. The standard sample (G 7041) was characterized by the lowest values of storage modulus in comparison with all samples obtained in this work from cod skin. Probably, such differences were due to the differences in molecular weight of the samples (see Table 4). The gelatin sample obtained without enzyme (G1) was characterized by the highest molecular weight—159 kDa.

It should be noted that there was a wide range of deformation amplitudes in which the linear viscoelastic behavior of the samples was observed (Figure 8a). The limit of linear viscoelastic behavior for G′ was in the range of deformation amplitude values from 80 to 200%.

Figure 8b shows the frequency dependences for gelatin samples obtained at 6 °C and a deformation amplitude of 1% corresponding to a region of linear viscoelastic behavior. Gelatin G1 had the highest values of storage modulus. In addition, a linear dependence of the storage modulus on frequency is observed over the entire frequency range. The linear nature of this dependence indicates that gelatin forms the most stable and rigid gel network [42,43]. The values of storage modulus for gelatin samples obtained using enzymes (G2 and G3) are close and comparable to the values obtained for the sample G 7041. However, the nature of these dependencies deviates from linear in the frequency range from 1 to 500 s^−1^. This behavior indicates that the intermolecular interactions of gelatin obtained using enzymes are weaker than those of gelatin obtained using acidic treatment.

**Figure 9:**
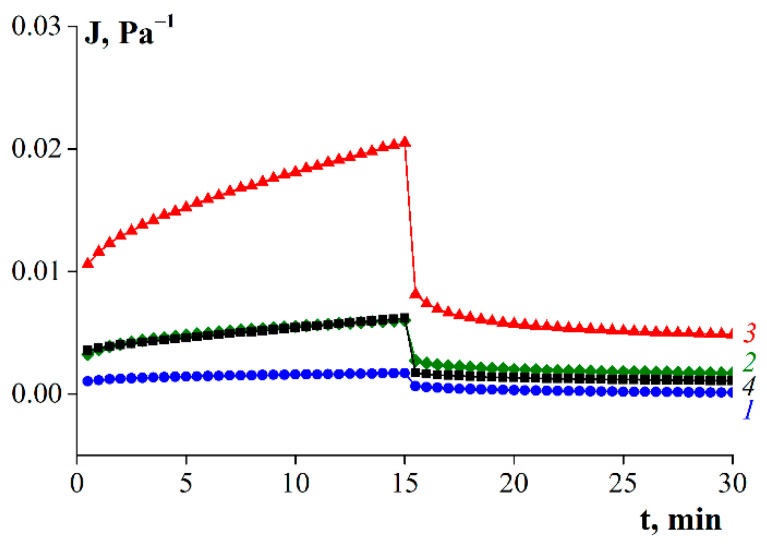
Compliance in loading (left side, t = 0–15 min) and recovery (right side, t = 15–30 min) with time at T = 6 °C and σ = 10 Pa, where: 1—G1, 2—G2, 3—G3, 4—G 7041. Gelatin concentration was 10 wt.%.

Figure 9 shows the evolution and recovery of deformation in time, obtained under loading (σ = 10 Pa, left side, 0–15 min) and unloading (σ = 0 Pa, right side, 15–30 min) of fish gelatin gels. A complete set of experimental data at different stresses is presented in Supplementary Files (Figure S1). The curves obtained during loading of the samples represent the dependence of the compliance on time, where J is the ratio of the deformations depending on the time at given stress:

(4) $$J(t,\;\sigma )=\frac{\gamma (t)\;}{\sigma }{=G}^{-1},$$

where γ—deformation; σ—shear stress; t—time, G—elastic modulus.

The later stages of the compliance curves obtained under loading (0–15 min) in Figure 8 show linear dependence with varying degrees of sloping. This behavior is typical for solid materials. However, when the load was removed, the residual deformations—which are characteristic of liquids—remained, and superimposed viscous effects appeared. Thus, gels obtained from fish gelatin are viscoelastic materials or so-called “soft matter”.

Figure 9 shows that at the same shear stress, the highest compliance values were observed for gelatin G3. Additionally, G3 was characterized by large values of residual deformations that persisted when the load was removed. The sample obtained without enzyme underwent the least deformation. The behavior of sample G2 was almost identical to that of sample G 7041.

Thus, the studied fish gelatin samples were characterized by a combination of reversible (elastic, γ_el_) and irreversible (viscous, γ_fl_) components of deformation. Based on this, the elastic modulus G can be defined as:

(5) $$G=\frac{\sigma }{\gamma_{\mathrm{el}}},$$

where γ_el_—residual deformation corresponding to the height of the plateau on the right-hand part of J(t) dependence. 

Dynamic viscosity η can be defined as:

(6) $$\eta =\frac{1}{\mathrm{dJ}/\mathrm{dt}}=\frac{1}{{d\gamma }_{\mathrm{fl}}/\mathrm{dt}},$$

where dγ_fl_/dt is determined by the steady slope in the left-hand part of J(t) dependence.

**Figure 10:**
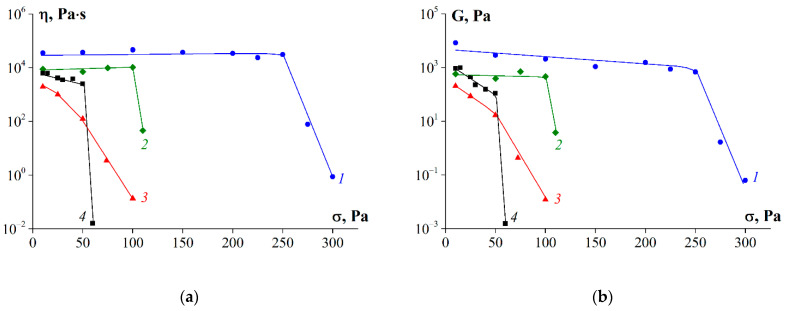
Dependence of (**a**) shear viscosity and (**b**) elastic modulus on shear stress at T = 6 °C, where 1—G1, 2—G2, 3—G3, 4—G 7041. Gelatin concentration was 10 wt.%.

Figure 10 shows the dependences of viscosity and elastic modulus on shear stress obtained at a temperature of 6 °C. Both rheological parameters had practically constant values for gelatin samples G1 and G2 for shear stresses up to 250 and 100 Pa, respectively. For other samples, this limit was less than 50 Pa. Sharp drops in the elastic modulus and viscosity at certain threshold stresses, shown in Figure 10, can be explained by the destruction of gels and are considered the limit strengths of the gelatins.

## 4. Discussion

The analysis presented in Table 2 shows that the use of commercial enzymes, even in small amounts (0.025 g/kg of raw material), can significantly reduce the time taken for the extraction of gelatin: 1.5 times compared with the standard extraction procedure at neutral pH values. The yield of the final product increased by almost 10% while the extraction time was reduced by 2 h. It is important to note that the chemical compositions of the gelatin samples remained practically unchanged (Table 2). Thus, the use of enzymes can reduce energy costs in the production of gelatin.

As can be seen in Table 4, enzyme preparations used at the stage of gelatin extraction affect the molecular weight of the final gelatin. The use of pancreatin led to a decrease in the average molecular weight from 159–97 kDa in comparison with the sample obtained without enzyme (Table 4). However, the molecular weight of gelatin obtained using protosubtilin (G2) was slightly higher than that of a commercial sample of fish gelatin, G 7041, considered as a standard sample. Pancreatin promotes more comprehensive denaturation of native collagen and can not only cleave hydrogen bonds between α-chains but also partially hydrolyze peptide bonds in the gelatin molecule.

**Figure 11:**
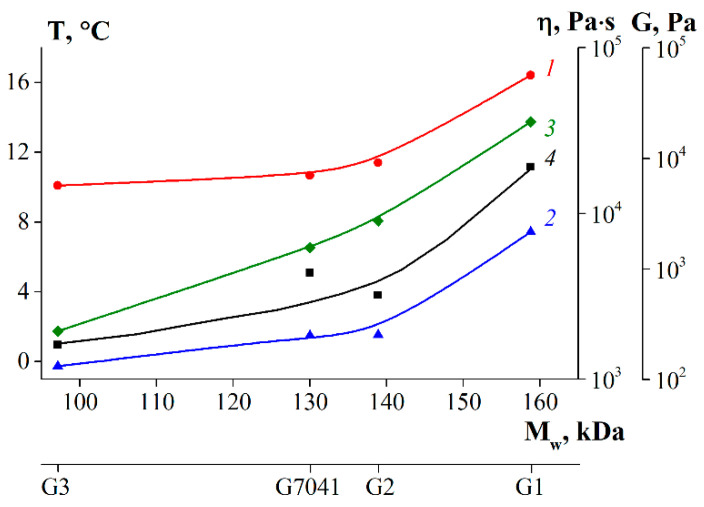
Dependencies of fish gelatin properties on molecular weight, where 1—T_m_, 2—T_g_, 3—η, 4—G.

The results obtained show correlations between the average molecular weight of fish gelatin and its properties. As the molecular weight increased (Table 4), the content of disordered structures decreased, and the triple helix content increased (Table 6). The structure of gelatin macromolecules also affects their thermal and rheological properties. Thus, the sample obtained without enzyme (G1) was characterized by the highest values of melting and gelation temperatures (Table 7) due to the highest content of ordered structures—triple helices. Triple helices play the role of additional nodes of the gel network formed during the cooling of the gelatin sol. The strength and rheological parameters (G′, G, η) of said gel (Figure 8 and Figure 10) were also an order of magnitude higher than those of other samples (G2, G3, G 7041). Gelatin obtained using pancreatin (G3) demonstrated the poorest rheological characteristics. It is interesting to note that the time and rate of gel formation for gelatin G3 were much higher than those of other samples (Figure 7). This discussion of the obtained results (Table 4, Table 6, Table 7, and Figure 10) is clearly presented in Figure 11. Gelatin samples G1 and G2 had characteristics (T_m_, T_g_, G, η) superior to those of sample G 7041 (Table 7 and Figure 10) due to the higher molecular weight and content of ordered structures (triple helices) (Table 4 and Table 6).

## 5. Conclusions

A comparative study of cod skin gelatin obtained by using enzymes with proteolytic action was conducted. The results indicate that the use of enzymes reduced the duration of extraction by 40%, and the yield of the final product was increased from 51% to 58–60%. Gelatin samples obtained using protosubtilin and pancreatin had lower average molecular weights (138.9 and 97.1 kDa, respectively) compared to sample obtained without enzymes (158.8 kDa). It was shown that as the molecular weight increased, the content of disordered structures decreased, and the triple helix content increased.

The systematic study of rheological properties of gels formed by fish gelatin was conducted. The sample obtained without enzyme was characterized by the highest values of melting and gelation temperatures and rheological properties (G′, G, η) due to the highest molecular weight and content of triple helices since triple helices play the role of additional nodes of the gel network. Gelatin obtained using pancreatin (G3) demonstrated the poorest rheological characteristics.

Thus, we can conclude that the use of protosubtilin is economically beneficial in reducing the extraction time. This technique can significantly increase the yield of gelatin whose quality and functional properties are similar to or superior to those of commercial fish gelatin. Furthermore, the proposed technique can reduce the number of reagents required for fish gelatin production, thereby promoting the development of environmentally sound technologies.

## Figures and Tables

**Table 1:** Names of gelatin samples from cod skin and their corresponding processing conditions.

Sample	Conditions
G1	Hydromodulus 1:4 pH = 7.0–7.5; T = 40 ± 1 °C; t = 5 h; without enzyme
G2	Hydromodulus 1:4 pH = 7.0–7.5; T = 40 ± 1 °C; t = 3 h; Enzyme—Protosubtilin (C_E_ = 0.025 g/1 kg of raw material)
G3	Hydromodulus 1:4 pH = 7.0–7.5; T = 40 ± 1 °C; t = 3 h; Enzyme—Pancreatin (C_E_ = 0.025 g/1 kg of raw material)

**Table 2:** Chemical composition of gelatin samples.

Sample	Moisture Content X, %	Total Nitrogen N_T_, %	Protein * P, %	Ash, %	Yield B, %
G1	4.8 ± 0.5	17.0 ± 0.1	94.4 ± 0.5	0.9 ± 0.1	51 ± 1
G2	5.6 ± 0.5	16.9 ± 0.1	93.8 ± 0.5	0.5 ± 0.1	60 ± 1
G3	6.8 ± 0.5	16.6 ± 0.1	92.0 ± 0.5	1.1 ± 0.1	58 ± 1
G 7041	5.5 ± 0.5	17.0 ± 0.1	93.5 ± 0.5	-	-

* Protein content was determined as P = N_total_·5.55 (5.55—conversion factor for nitrogen to collagen) [34].

**Table 3:** Amino acid compositions of gelatin samples.

Amino Acid	Abbreviation	Content, g/100 g				
		G1	G2	G3	Cod Skin	G 7041
Glycine	GLY	19.0	18.5	18.2	18.4	18.6
Proline	PRO	8.0	7.9	7.6	8.3	12.9
Alanine	ALA	10.0	9.1	9.6	9.3	9.4
Glutamic acid	GLU	10.4	10.7	10.0	10.0	9.3
Arginine	ARG	10.9	11.0	10.3	10.0	7.6
Aspartic acid	ASP	6.8	7.1	6.7	7.1	5.6
Serine	SER	6.3	6.4	6.0	6.0	6.3
Leucine *	LEU	3.3	3.4	3.3	3.8	2.8
Threonine *	THR	2.7	2.8	2.6	2.8	2.6
Phenylalanine *	PHE	2.3	2.3	2.2	2.4	2.4
Lysine *	LYS	4.6	4.8	4.6	4.5	2.3
Valine *	VAL	2.2	2.4	2.3	2.7	2.1
Methionine *	MET	0.8	0.7	0.7	1.8	1.6
Isoleucine *	ILE	1.8	1.8	1.7	1.9	1.5
Tyrosine *	TYR	0.9	0.8	0.9	1.2	0.8
Histidine *	HIS	1.5	1.2	1.6	1.6	1.7
Total		91.5	90.9	88.3	91.8	87.5

* Essential amino acids.

**Table 4:** Molecular weight distributions of gelatin samples obtained by HPLC.

Sample	M_wf_, kDa	ω, % *	M_w_, kDa **
G1	72.4	35.9	158.8
	207.2	64.1	
G2	45.2	39.0	138.9
	198.8	61.0	
G3	45.2	53.6	97.1
	157.1	46.4	
G 7041			131.5

M_wf_—molecular weight of fraction; * ω—proportion of each fraction (determined using the Gaussian distribution); ** M_W_—calculated by the additivity rule. Date are means ± standard deviation (n = 3). Different superscript letters in the same column indicate significant differences (*p* < 0.05).

**Table 5:** Main adsorption bands of the functional groups of gelatin [36,37].

Group	Wave Number ν, cm^−1^	Type of Vibration
Amide A	3400–3300	N–H stretching vibrations
Amide B	3000–2900	N–H stretching vibrations
Amide I	1700–1600	C=O stretching vibrations—80%, and C–N stretching vibrations
Amide II	1575–1480	N–H deformation vibrations—80%, and C–N stretching vibrations
Amide III	1300–1230	C–N stretching vibrations

**Table 6:** Contents (%) of major components of the secondary structure of gelatin samples.

Secondary Structure Element	Wave Number ν, cm^−1^	G1	G2	G3	G 7041
β-turn/β-sheet	1620–1624	8.3	12.1	10.4	13.6
	1635–1637	22.4	16.8	20.0	20.7
Random coil	1651–1653	17.2	21.1	28.7	23.1
Triple helix	1668–1670	42.9	40.6	22.4	24.2
β-turn/β-sheet	1686–1690	9.2	9.4	18.5	18.4

**Table 7:** Thermal properties of gelatin gels. Gelatin concentration was 10 wt.%.

Sample	T_m_, °C	T_g_, °C
G1	16.4	7.4
G2	11.4	1.5
G3	10.1	−0.3
G 7041	10.7	1.5

## Data Availability

Not applicable.

## Supplementary Materials

The following supporting information can be downloaded at: https://www.mdpi.com/article/10.3390/polym14040751/s1, Figure S1: Creep and recovery curves obtained at different stresses.
