# Supplementary material for: Characterization of Fish Gelatin Obtained from Atlantic Cod Skin Using Enzymatic Treatment
Source: Polymers (Basel). 2022 Feb 15;14(4):751. doi: 10.3390/polym14040751 (PMC8879374; doi:10.3390/polym14040751)
Supplement: Supplementary file 1 [file polymers-14-00751-s001.zip › polymers-1585156-supplementary.pdf]

Supplementary information

S1: Creep and recovery curves obtained at different stresses

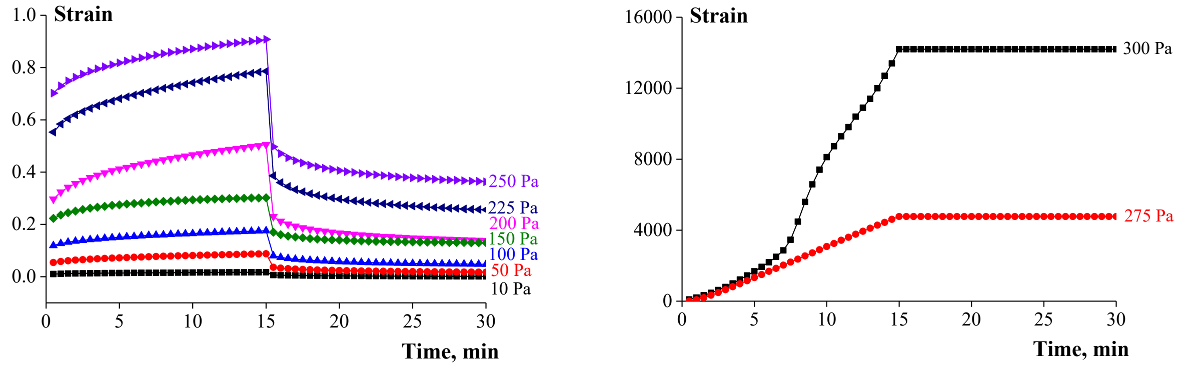

(a)

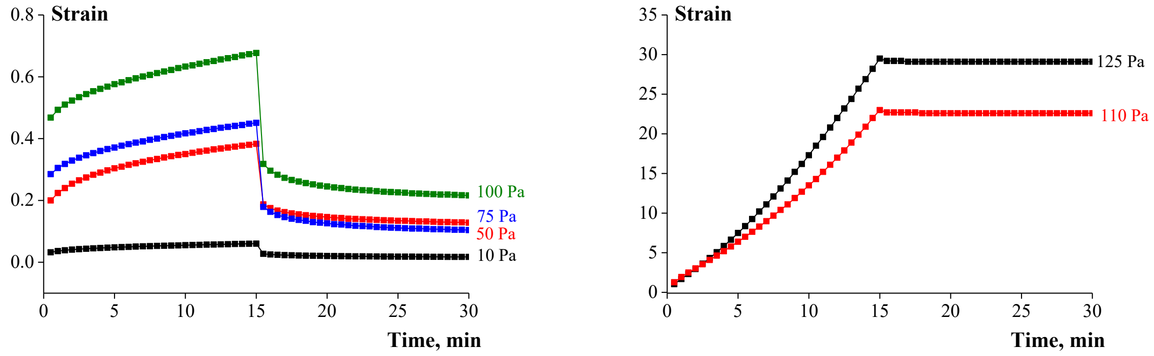

(b)

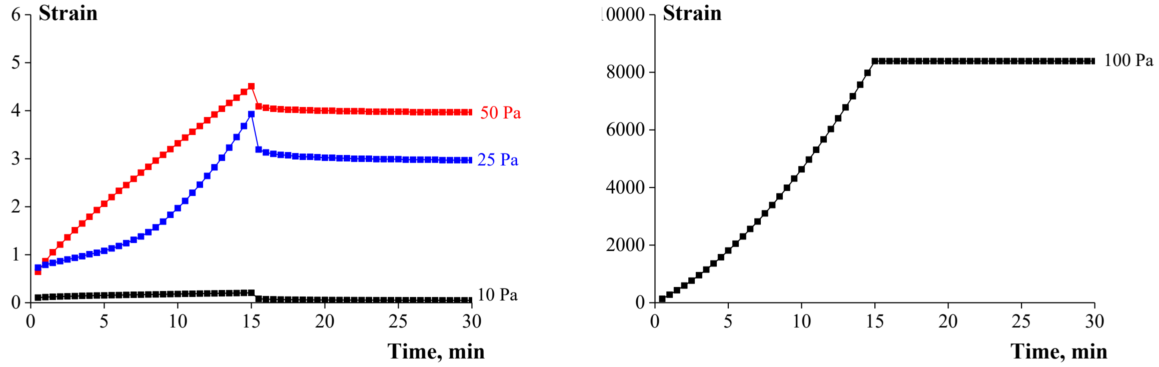

(c)

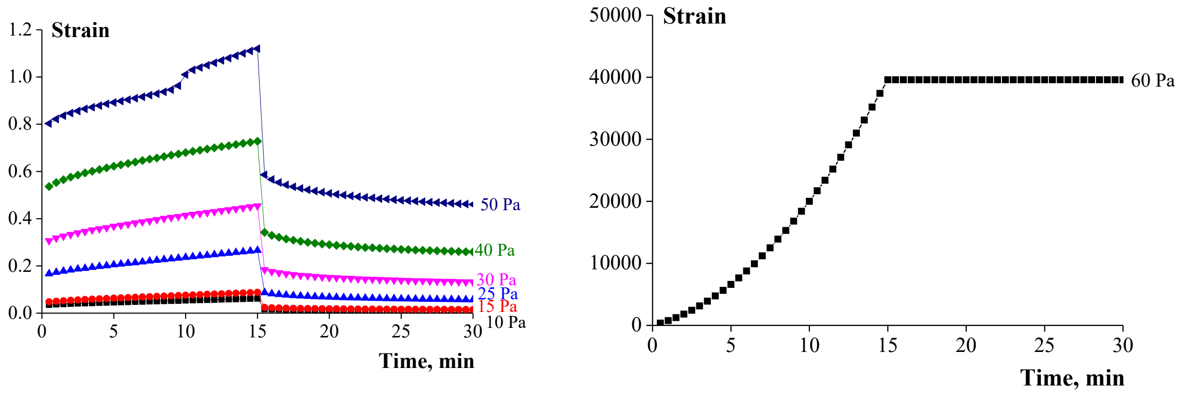

(d)

Figure S1. Compliance in loading (left side, t = 0–15 min) and recovery (right side, t = 15–30 min) with time at T = 6 °C and different stresses (σ, Pa), where: a—G1; b—G2; c—G3; d—G 7041. Gelatin concentration was 10 wt.%.
